# PHA-4/FOXA-regulated microRNA feed forward loops during *Caenorhabditis elegans* dietary restriction

**DOI:** 10.18632/aging.100697

**Published:** 2014-10-31

**Authors:** Awadhesh Pandit, Vaibhav Jain, Neeraj Kumar, Arnab Mukhopadhyay

**Affiliations:** Molecular Aging Laboratory, National Institute of Immunology, Aruna Asaf Ali Marg, New Delhi 110067, India

**Keywords:** microRNA, dietary restriction, PHA-4/FOXA, Transcription factor, miRNA, feed forward loops, aging

## Abstract

Dietary restriction (DR) increases life span and delays the onset of age-related diseases across species. However, the molecular mechanisms have remained relatively unexplored in terms of gene regulation. In *C. elegans*, a popular model for aging studies, the FOXA transcription factor PHA-4 is a robust genetic regulator of DR, although little is known about how it regulates gene expression. We profiled the transcriptome and miRNAome of an *eat-2* mutant, a genetic surrogate of DR, by Next Generation sequencing and find that most of the miRNAs are upregulated in the young-adult worms, none significantly downregulated. Interestingly, PHA-4 can potentially regulate the expression of most of these miRNA genes. Remarkably, many of the PHA-4-regulated genes that are induced during DR are also targets of the PHA-4-upregulated miRNAs, forming a large feed-forward gene regulatory network. The genes targeted by the feed-forward loops (FFLs) are enriched for functions related to ubiquitin-mediated decay, lysosomal autophagy, cellular signalling, protein folding etc., processes that play critical roles in DR and longevity. Together our data provides a framework for understanding the complex and unique regulatory network employed during DR, suggesting that PHA-4 employs such FFLs to fine-tune gene expression and instil robustness in the system during energy crisis.

## INTRODUCTION

Dietary restriction (DR) is the only intervention that can consistently increase life span and delay onset of age-related diseases in most of the model systems tested [1]. Since the initial discovery in 1935 [2] that animals fed less food lived substantially longer, DR has been shown to increase life span in many vertebrates [1, 3]. In several non-vertebrates that are important models for ageing research, like yeast, *Drosophila* and *Caenorhabditis elegans*, DR increases life and health span. In mammals, DR significantly protects against age-related disorders like type II diabetes, atherosclerosis and Alzheimer's disease [1]. Although a large body of literature exists about the physiological consequences of DR, the molecular mechanisms by which DR increases longevity are still unclear [4].

Invertebrate models like *C. elegans* have provided tremendous insight into the mechanisms of aging and longevity assurance in general, and DR in particular. DR is achieved through multiple genetic and extrinsic interventions in worms [5-8]. Mutations in an acetylcholine receptor gene, *eat-2* lead to decreased pharyngeal pumping resulting in lowered food intake [6]. The *eat-2* model of DR remains the most-studied genetic surrogate used to decipher the mechanisms associated with the process, although DR-like phenotypes can be obtained by other gene perturbations [9, 10]. DR can also be initiated by diluting the bacterial food that the worms ingest [5, 11]. Interestingly, life span extension on DR brought about by both genetic and non-genetic interventions depends upon the function of a conserved FOXA transcription factor, PHA-4 [11]. The molecular mechanisms explaining how PHA-4 mediates DR-induced longevity are currently less clear.

MicroRNAs (miRNAs) have emerged as important and conserved gene expression modulators across phylogeny [12]. Primary miRNAs are transcribed in the nucleus and processed by the Drosha complex to produce precursor miRNA (pre-miRNA, ~70 nucleotides long) [13, 14]. These pre-miRNAs are exported out of the nucleus, by the Exportin complex, into the cytoplasm where they are processed by the Dicer enzyme to generate mature miRNA [13, 14]. The mature miRNA associates with specific Argonaute proteins leading either to the degradation of target mRNA or attenuation of translation, depending on the homology of its seed region with the 3′-UTR of the mRNA [13-16]. MiRNA-mRNA interactions have been proposed to shield protein expression from various oscillations in transcription during development, thereby installing robustness in the system that prevents phenotypic fluctuations- a process called canalization [17-22]. Although contributions of miRNA have been documented in many biological processes in *C. elegans* and other organisms, including aging [23-28], little is known about their role in dietary restriction.

In an effort to study the transcriptional and post-transcriptional response to DR in details, we profiled the mRNA and miRNA of *eat-2(ad1116)* by Next Generation Sequencing (NGS). Here we show that many of the miRNAs (82 out of 368) are upregulated in the day 1 young-adult mutant worms. Remarkably none are significantly downregulated. This pattern was unique to *eat-2* mutation-mediated DR as it was significantly different from the profile of low insulin signalling mutant, *daf-2(e1370)*. Using published ChIP-seq data for PHA-4 [29, 30] and qRT-PCR validation, we show that 65 of the 82 miRNAs are potential direct transcriptional targets of PHA-4. The transcriptomics followed by bioinformatics analysis and qRT-PCR validation showed that PHA-4 may regulate a large portion of the transcripts that are upregulated in *eat-2(ad1116)*. Interestingly, the PHA-4-regulated miRNAs target a major portion of the PHA-4-regulated transcripts during DR, thereby forming transcription factor incoherent feed forward loops (FFLs). The FOXO transcription factor DAF-16 does not appear to extensively utilize such FFLs during conditions of low insulin signalling. We hypothesize that these FFLs modulate the expression of PHA-4-dependent genes during energy-depleted conditions, building robustness into the system that reduce phenotypic fluctuations. These genes are involved in ubiquitin-mediated protein degradation, lysosomal autophagy, protein folding, signalling as well as metabolism, processes that need to be strongly regulated during energy crisis. Thus, our study elucidates the complexity of gene regulation following initiation of DR in *eat-2(ad1116)* and defines the central role of FOXA/PHA-4 in this process, justifying its position as a robust genetic regulator of DR-induced longevity.

## RESULTS

### Next generation sequencing reveals *eat-2(ad1116)* miRNA profile

In order to gain an insight into the complex interplay of miRNAs during DR, we performed NGS analysis to compare miRNA profiles of wild-type (WT) N2 Bristol and *eat-2(ad1116)*. The *eat-2(ad1116)* strain lives considerably longer than WT; however, none of the strains have significant mortality at day 1 (equivalent to young-adults) or day 8 of adulthood (aging worms) (Figure S1). The *eat-2* mutants have defective pharyngeal pumping and consume less food since hatching. We hypothesized that the system in *eat-2(ad1116)* is already geared up for long life and would have significantly modified post-transcriptional cellular environment on day 1 of adulthood i.e., at the young-adult stage. Additionally, we wanted to study the changes that occur later in their life, as compared to WT, in order to understand the late-life cellular response to DR. Synchronized worms were harvested on day 1 or 8 of adulthood and small RNA sequencing libraries prepared (see experimental procedures). Each sample was run in a single lane of a flow cell in the Genome Analyzer *IIx* (GA*IIx*) platform (Illumina Inc., USA) that led to the generation of around 20 million reads per lane with an average trimmed read size of ~22 nucleotides (Figure S2A). All reads mapped to the *C. elegans* genome while 81-95% readily mapped to the miRBase release 19 that has a collection of 368 worm miRNAs (Figure S2B, 1). We detected 185 miRNA in day 1/young-adult worms and 187 in day 8/aging WT samples, while 224 and 161 miRNA were detected in *eat-2(ad1116)* samples in day 1 and day 8 samples, respectively, all miRNA having greater than 10 read counts (Table S1). We were also able to detect between 0.5-1.2 million reads that mapped to known 21U-RNAs (Table S1). After eliminating rRNA, tRNA and ncRNA reads, the remaining un-annotated reads were processed for novel miRNA discovery, as discussed below.

We compared the miRNA profiles of WT worms collected at days 1 or 8 with a previously published study on changes in miRNA profile during aging in WT nematode [31] (Table S2). Out of a total of 21 miRNA reported to be upregulated in day 8 compared to day 1 in their study, 16 matched to our data. We found an additional 20 miRNA upregulated in day 8 samples. When we compared the downregulated miRNAs from the two studies, 7 out of 18 matched our data while we obtained an additional 24 miRNAs, probably because of increased depth of sequencing. It may also be due to the less number of miRNAs (174) present in the miRbase release 14, to which the earlier data was mapped. Together, the sequencing depth and coverage attained in our study includes most of the known miRNAs and has the potential to discover new and low expressing ones.

### Unique changes in miRNA profile in *eat-2(ad1116)* worms

**Figure 1:**
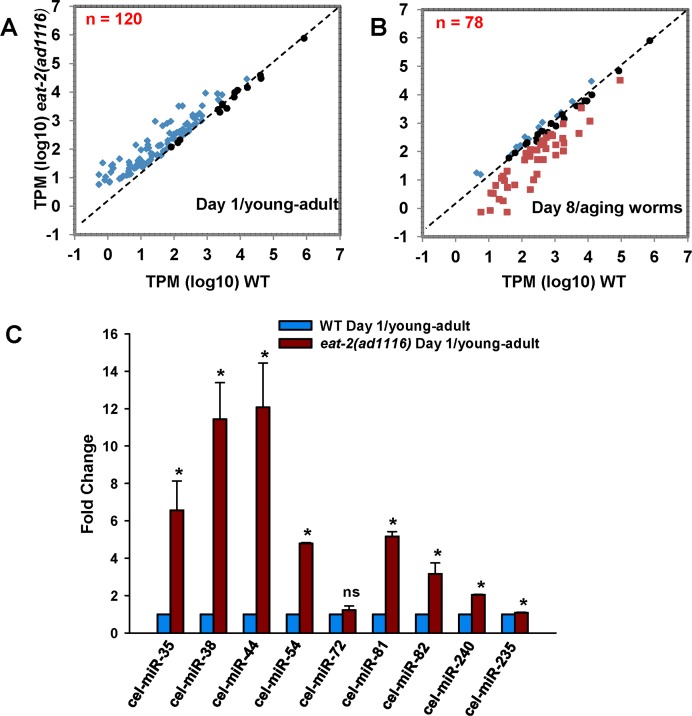
Differential regulation of miRNA population in WT and *eat-2(ad1116)*. A scatter plot of miRNAs commonly expressed between WT and *eat-2(ad1116)* in (**A**) day 1 young-adult worms or (**B**) aging day 8 worms. For this, we considered 120 (day 1) or 78 (day 8) miRNAs (see Table S3) that changed expression in *eat-2(ad1116)* as compared to WT and had read counts >10. (**C**) Validation of sequencing results by quantitative real-time PCR. The miRNA expresion of young-adult *eat-2(ad1116)* was compare to WT worms collected on day 1 of adulthood. Error bars indicate standard deviation between independent biological replicates. The p-values were calculated by*t*-test (* represent *p* < 0.05).

We compared the normalized miRNA expression (TPM, see experimental procedures) of WT with *eat-2(ad1116)* either in day 1/young-adults or in day 8/aging worms and found that most of the miRNAs were distinctly upregulated in young-adult *eat-2(ad1116)* (Figure 1A, Figure S3 for analysis flow chart, Table 1, Table S3). For this, we considered all miRNAs that had read counts over 10 in both the strains on a particular day, with minimum fold change of ±1.5 having a *p*<0.05. Out of a total of 184 miRNA common between young-adult WT and *eat-2(ad1116)* on day 1, 105 were significantly upregulated in *eat-2(ad1116)*. Interestingly, none were significantly downregulated. Seventy nine miRNA had no significant change in expression pattern. We found that 40 miRNA were exclusively expressed in *eat-2(ad1116)* (Table S4); out of them *cel-miR-41-5p*, *cel-miR-40-5p*, *cel-miR-39-5p*, *cel-miR-4813-5p* had read counts more than 100. For further bioinformatic analysis using these upregulated miRNAs in day 1/young-adult worms, we did not consider the strandedness of the miRNAs and only analysed 82 out of the 105 as uniquely upregulated miRNAs.

**Table 1:** List of miRNAs that were significantly upregulated in young-adult/Day 1 *eat-2(ad1116)* worms as compare to WT (more details in Table S3)

S. No.	miRNA	Fold Change	*p*-value	S. No.	miRNA	Fold Change	*p*-value
**1**	***cel-miR-82-5p***	**47.46**	<0.0001	54	*cel-miR-253-3p*	3.3	<0.0001
2	*cel-miR-38-3p*	35.19	<0.0001	55	*cel-miR-237-3p*	3.29	0.0300
3	*cel-miR-74-5p*	23.95	0.0008	56	*cel-miR-78*	3.22	0.0039
**4**	***cel-miR-54-5p***	**23.41**	<0.0001	57	*cel-miR-784-5p*	3.16	<0.0001
**5**	***cel-miR-44-5p***	**20.61**	<0.0001	**58**	***cel-miR-240-3p***	**3.15**	<0.0001
6	*cel-miR-35-5p*	20.59	<0.0001	59	*cel-miR-73-3p*	3.12	<0.0001
**7**	***cel-miR-81-5p***	**20.33**	<0.0001	60	*cel-miR-58-5p*	3.07	<0.0001
8	*cel-miR-229-3p*	19.88	<0.0001	61	*cel-miR-77-3p*	3.07	<0.0001
9	*cel-miR-35-3p*	18.36	<0.0001	62	*cel-miR-85-3p*	3.05	<0.0001
10	*cel-miR-73-5p*	14.88	<0.0001	63	*cel-miR-43-3p*	3.03	<0.0001
11	*cel-miR-36-3p*	14.83	<0.0001	64	*cel-miR-787-3p*	2.93	0.0400
12	*cel-miR-41-3p*	14.65	<0.0001	65	*cel-miR-60-3p*	2.88	<0.0001
13	*cel-miR-39-3p*	13.03	<0.0001	**66**	***cel-miR-235-3p***	**2.87**	<0.0001
14	*cel-miR-40-3p*	12.78	<0.0001	67	*cel-miR-786-3p*	2.78	0.0400
15	*cel-miR-255-3p*	12.6	0.0005	68	*cel-miR-80-5p*	2.73	<0.0001
16	*cel-miR-79-5p*	12.42	0.0038	69	*cel-miR-785*	2.72	<0.0001
17	*cel-miR-37-3p*	11.92	<0.0001	70	*cel-miR-63-3p*	2.71	<0.0001
18	*cel-miR-66-3p*	10.85	0.0400	71	*cel-miR-49-3p*	2.7	<0.0001
19	*cel-miR-37-5p*	10.67	0.0400	72	*cel-miR-1817*	2.68	0.0060
**20**	***cel-miR-72-5p***	**10.45**	<0.0001	73	*cel-miR-231-3p*	2.64	<0.0001
21	*cel-miR-64-3p*	10.18	0.0100	74	*cel-miR-66-5p*	2.54	<0.0001
22	*cel-miR-71-3p*	9.43	<0.0001	75	*cel-miR-74-3p*	2.45	<0.0001
23	*cel-miR-77-5p*	8.72	<0.0001	76	*cel-miR-51-5p*	2.3	<0.0001
24	*cel-miR-252-5p*	8.08	<0.0001	**77**	***cel-miR-47-3p***	**2.29**	<0.0001
25	*cel-miR-238-5p*	7.07	<0.0001	78	*cel-miR-359*	2.24	0.0100
26	*cel-miR-83-5p*	6.53	0.0300	79	*cel-miR-64-5p*	2.23	<0.0001
27	*cel-miR-83-3p*	6.43	<0.0001	80	*cel-miR-34-5p*	2.22	0.0100
28	*cel-miR-56-5p*	6.17	<0.0001	81	*cel-miR-793*	2.19	<0.0001
29	*cel-miR-72-3p*	6.07	0.0400	82	*cel-miR-54-3p*	2.19	0.0064
30	*cel-miR-52-3p*	5.03	<0.0001	83	*cel-miR-259-5p*	2.16	0.0037
31	*cel-miR-42-3p*	5.03	<0.0001	84	*cel-miR-34-3p*	2.08	0.0400
32	*cel-miR-84-3p*	4.88	0.0008	85	*cel-miR-355*	2.08	0.0006
33	*cel-miR-228-3p*	4.87	<0.0001	86	*cel-miR-239a-5p*	2.01	<0.0001
34	*cel-miR-241-5p*	4.74	<0.0001	87	*cel-miR-1018*	1.95	0.0019
35	*cel-miR-71-5p*	4.37	<0.0001	88	*cel-miR-86-3p*	1.89	0.0036
36	*cel-miR-238-3p*	4.32	<0.0001	89	*cel-miR-1-3p*	1.85	<0.0001
37	*cel-miR-4937*	4.07	0.0400	90	*cel-miR-248*	1.84	0.0006
38	*cel-miR-70-5p*	4.07	<0.0001	91	*cel-miR-46-3p*	1.82	<0.0001
39	*cel-miR-1829b*	4.04	0.0052	**92**	***cel-miR-246-3p***	**1.81**	<0.0001
40	*cel-miR-75-3p*	3.96	<0.0001	93	*cel-miR-65-5p*	1.8	<0.0001
41	*cel-miR-45-5p*	3.95	0.0200	94	*cel-miR-229-5p*	1.73	<0.0001
42	*cel-miR-75-5p*	3.94	0.0001	95	*cel-miR-56-3p*	1.72	<0.0001
43	*cel-miR-47-5p*	3.8	0.0005	96	*cel-miR-87-3p*	1.71	0.0002
44	*cel-miR-358-3p*	3.79	0.0400	97	*cel-miR-254*	1.70	<0.0001
45	*cel-miR-1-5p*	3.69	0.0001	98	*cel-miR-243-3p*	1.70	0.0010
46	*cel-miR-67-3p*	3.66	<0.0001	99	*cel-miR-1830-3p*	1.69	<0.0001
47	*cel-miR-240-5p*	3.62	<0.0001	100	*cel-miR-61-3p*	1.68	<0.0001
48	*cel-miR-239b-5p*	3.55	<0.0001	101	*cel-miR-79-3p*	1.68	<0.0001
49	*cel-miR-230-5p*	3.55	0.0002	102	*cel-miR-236-3p*	1.61	<0.0001
50	*cel-miR-51-3p*	3.5	<0.0001	103	*cel-miR-86-5p*	1.51	<0.0001
51	*cel-miR-5592-5p*	3.4	<0.0001	104	*cel-miR-244-5p*	1.50	<0.0001
52	*cel-miR-5592-3p*	3.4	0.0041	105	*cel-miR-2-3p*	1.50	0.0500
53	*cel-miR-55-3p*	3.32	<0.0001				

MiRNAs that were used for QRT-PCR validation are in bold.

MiRNAs that did not possess any PHA-4 binding peaks are indicated in red. All the other miRNAs had one or more binding peaks within 5 kb upstream and 1 kb downstream of the transcription start site.

In order to validate the sequencing results, we chose 9 miRNAs having varying expression levels. Using quantitative real time PCR, we could validate the expression pattern of eight of these miRNAs; they were significantly upregulated in *eat-2(ad1116)* compared to WT on day 1 (Figure 1C).

Next we compared the miRNA profiles of aging WT and *eat-2(ad1116)* worms collected at day 8 of adulthood (Figure 1B, Table S3). We found 157 miRNAs common between WT and *eat-2(ad1116)*; 15 were significantly upregulated in *eat-2(ad1116)* while 43 were downregulated compared to WT. Twenty miRNAs had no significant change in expression pattern. Four miRNAs were exclusively expressed in *eat-2(ad1116)* while 30 were present only in WT; out of them 4 had read counts more than 100 (Table S5).

**Figure 2:**
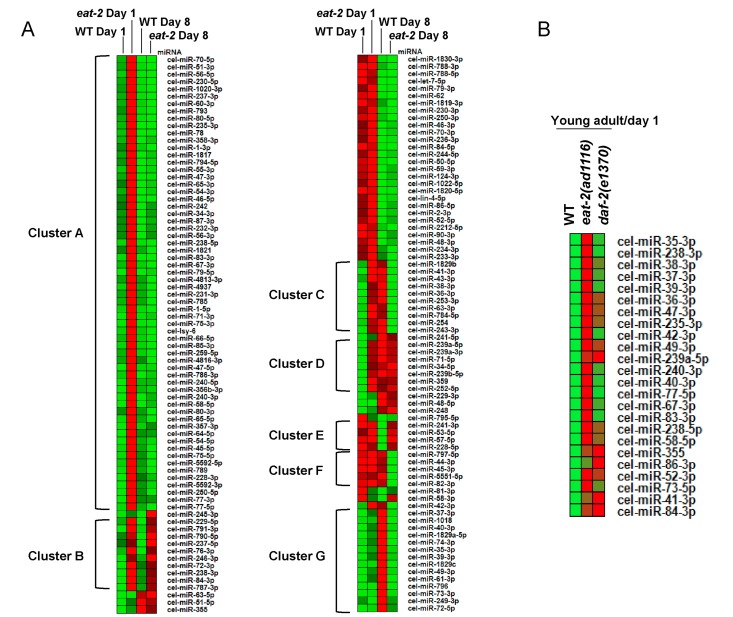
Age-dependent changes in miRNA expression. (**A**) A Heatmap showing expression patterns of 152 miRNAs that are commonly expressed in WT and *eat-2(ad1116)* at day 1 (young-adults) and day 8 (aging worms) of adulthood. The miRNAs are grouped into distinct clusters A-G based on the similarity in their expression patterns. (**B**) A Heatmap comparing the expression of miRNAs in WT, *eat-2(ad1116)* and *daf-2(e1370)*.

From the above observations, it appeared that many miRNAs that are upregulated in young-adult *eat-2(ad1116)* on day 1 returned to the levels of WT or lower with progressing age. When we compared the expression patterns of miRNAs that were expressed in all the four samples with a minimum read count of 10, we found that 61 of the 152 (~40%) were upregulated in *eat-2(ad1116)* on day 1 but returned to near WT levels on day 8 (Cluster A in Figure 2A; Table S6). These included miRNAs *cel-miR-70*, *cel-miR-51*, *cel-miR-56*, *cel-miR-230*, *cel-miR-238*, *cel-miR-83*, etc. There were 10 others that were upregulated in day 1 of *eat-2(ad1116)*, but remained upregulated even in day 8 (Cluster B). These included miRNAs *cel-miR-237*, *cel-miR-238*, *cel-miR-229*, *cel-miR-791* and *cel-miR-72*. Levels of miRNAs *cel-miR-241*, *cel-miR-53*, *cel-miR-57* and *cel-miR-228* were decreased in day 8 in WT compare to day 1 levels, but failed to so in *eat-2(ad1116)* (Cluster E). Several miRNAs had expression patterns that were reverse of that in WT worms. For e.g., 11 miRNA, including *cel-miR-63*, *cel-miR-53*, *cel-miR-36*-*41*, *cel-miR-254* and *cel-miR-61* increased in expression from day 1 to 8 in WT worms; however, in *eat-2(ad1116)* it had an inverse expression pattern (Cluster C). As a corollary, only *cel-miR-58* had the opposite expression pattern-decreased in WT but increased in *eat-2(ad1116)*. Apart from miRNAs expressing in all samples, another 79 miRNA had more than 10 read count in any one or more of the 4 samples and were categorized separately (Table S7). Most of these miRNAs had low abundance as indicated by their read counts that were less than 1000. Only *cel-miR-86* had 1258 read counts (TPM 58.72) on day 1 but had no detectable count on day 8. In this list also, we found 29 miRNAs were exclusively expressed in *eat-2(ad1116)* on day 1. These observations point to the profound changes in miRNA expression during *eat-2(ad1116)-*mediated dietary restriction, possibly to support metabolic changes associated with the long life span. MicroRNAs were, in general, found to be upregulated in *eat-2(ad1116)* young adult worms on day 1 of adulthood.

### The miRNAome of *eat-2(ad1116)* worms is different from the long-lived insulin-like signalling mutant

Next we asked whether the profile obtained with *eat-2(ad1116)* is a general pattern for all long-lived mutants. In worms, the insulin-like signalling pathway is a major regulator of longevity and development [32]. Mutations in the insulin-like receptor *daf-2* leads to dramatic increase in life span [33]. However, the mechanisms by which DR and insulin-like signalling increase life span are independent of each other [6]. We profiled the miRNAome of *daf-2(e1370)* at day 1 of adulthood and found that only 24 miRNAs were upregulated significantly while 33 were downregulated (Table S1, S18). All 24 upregulated miRNAs were also commonly detected in *eat-2(ad1116)* young adult worms, although the levels were higher in the latter in most cases (Figure 2B). In stark contrast to *daf-2(e1370)*, none of the miRNAs were downregulated in *eat-2(ad1116)* as mentioned above. Together these data showed that the profile of miRNA in *eat-2(ad1116)*-mediated DR worms is unique and does not represent a common trend of long-lived mutants. However, some of the commonly upregulated miRNAs may still correspond to a signature for long-lived mutants, but needs to be validated in other strains that have enhanced longevity.

### Novel miRNA that change expression in *eat-2(ad1116)*

Since DR induces a dramatic reprogramming of development and metabolism, we hypothesized that novel miRNAs may contribute towards this effect. Additionally, with increased depth of sequencing, we expected to detect novel miRNAs in our WT samples also. We focused our attention on the 877496 (in young adults on day 1) and 682280 (in day 8/aging worms) non-redundant un-annotated reads that aligned to the *C. elegans* genome in *eat-2(ad1116)* (Table S1). In order to discover novel miRNAs, we used the miRNA discovery package miRDeep2 (see Supporting information for reference). We found 48 novel miRNAs in young-adults and 9 in aging *eat-2(ad1116)* worms, with a read count greater than 10. Additionally following analysis of unassigned reads, in WT we identified 21 in young-adults and 35 in aging worms (8 and 9). Out of the 13 commonly predicted miRNAs between day 1/young-adult WT and *eat-2(ad1116)* (without read count or *p* value cut-off) worms, 9 were upregulated and 3 down-regulated as calculated on the basis of read count (Table S10). On the other hand, 2 out of 3 common miRNAs on day 8 were downregulated in *eat-2(ad1116)*. Thus, similar to the known miRNA, we found distinct upregulation of novel miRNA population in young-adult *eat-2(ad1116)* worms.

**Figure 3:**
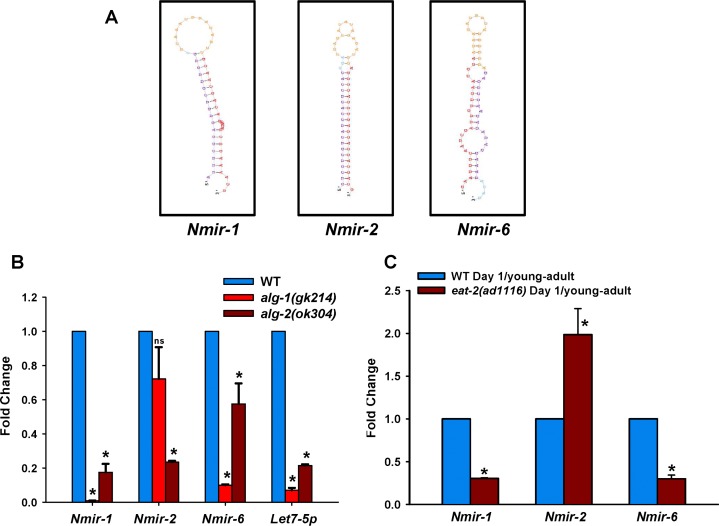
Expression of novel miRNAs during DR. (**A**) The secondary structures of 3 predicted novel miRNA candidates as generated using the miRDeep2 program. (**B**) Expression of predicted novel miRNAs were examined in specific Argonaut gene mutants *alg-1(gk214)* or *alg-2(ok304)* by QRT-PCR (**C**) Expression changes of novel miRNAs in *eat-2(ad1116)* as compare to WT collected on day 1 of adulthood (young-adult worms) by qRT-PCR. Error bars indicate Standard Deviation between independent biological replicates. The *p*-values were calculated by *t*-test (* represents *p* < 0.05).

In order to select predicted novel miRNA for experimental validation, we used one additional criterion. Apart from read count filter of ≥10 and presence of a star sequence, we used MiRDeep Score >1 as a cut-off. Using these criteria, we identified 7 predicted miRNA in WT and 15 in *eat-2(ad1116)* young-adult worms on day 1, and 5 in aging WT as well as 1 in aging *eat-2(ad1116)*. From these, we selected 3 predicted novel miRNA for validation (Figure 3A, Table S11). We grew WT, *alg-1(gk214)* or *alg-2(ok304)* and checked the expression of the predicted miRNAs at young-adult stage. The ALG-1 and ALG-2 proteins are required for miRNA maturation [34]. We found that all of the candidate miRNAs expressed in an *alg-2*-dependent manner while 2 expressed in *alg-1*-dependent manner as determined by quantitative real-time PCR analysis (Figure 3B), confirming that they are indeed *bona fide* miRNAs. Out of these three novel miRNA, two are down-regulated in *eat-2(ad1116)* young-adult worms while one is upregulated when compared to WT (Figure 3C), matching their expression as determined by sequencing. Together, it appears that the profound transcriptional and post-transcriptional changes required following DR in *eat-2(ad1116)* involves the role of additional novel miRNAs.

### PHA-4 regulates majority of the upregulated miRNA in *eat-2(ad1116)*

**Figure 4:**
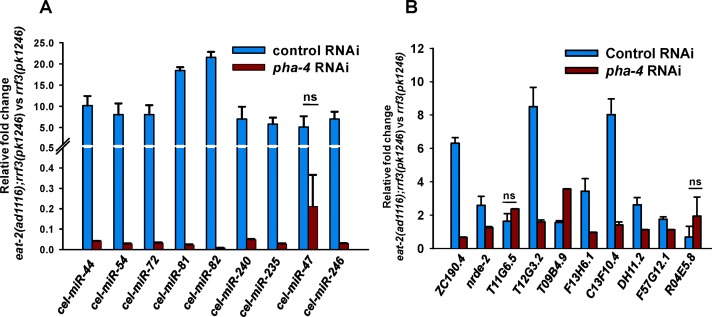
The miRNAs and mRNAs upregulated during DR are potential direct targets of PHA-4. (**A**) QRT-PCR analysis shows the expression levels of the miRNAs in *eat-2(ad1116);rrf-3(pk1246)* compared to *rrf-3(pk1246)*, when grown on control or pha-4 RNAi. (**B**) QRT-PCR analysis showing the expression levels of predicted PHA-4 direct target genes in *eat-2(ad1116);rrf-3(pk1246)* compared to *rrf-3(pk1246)*, grown either on control and *pha-4* RNAi. The error bars represent Standard Deviation between independent biological samples. ‘ns’ represents *p* ≥ 0.05 by *t*-test.

The FOXA transcription factor PHA-4 is an absolute requirement for DR-induced longevity in *eat-2* mutants [11]. We asked whether PHA-4 could be directly regulating the expression of the miRNAs upregulated in *eat-2(ad1116)*. For this, we used ChIPBase (See Supporting information for reference), a database that catalogues transcription factor binding maps on miRNA (as well as other small RNAs) and protein-coding gene promoters using ChIP-seq data from published work [29, 30]. We queried the database with all the miRNAs that were upregulated in young-adult *eat-2(ad1116)* worms collected on day 1. We found that 65 of the 82 promoters of upregulated miRNA have one or more PHA-4 binding peaks (Table 1), potentially pointing at direct transcriptional regulation of these miRNA by PHA-4. In order to validate this data, we chose 9 of these miRNAs that are induced in *eat-2(ad1116)* as compared to WT and checked their expression in *eat-2(ad1116)* mutants, both in presence or absence of PHA-4. We found that all these miRNAs, except *cel-mir-47*, are regulated in a PHA-4-dependent manner (Figure 4A). Thus, PHA-4 not only controls mRNA levels of coding genes, like *sod-2* and *sod-4* during DR [11], it possibly regulates the transcription of a large part of the miRNA population.

### DR-induced miRNAs in *eat-2(ad1116)* may target a large part of the transcriptome

We discovered dramatic changes in miRNA expression levels with DR in *eat-2(ad1116)*. We also identified PHA-4 as a transcription factor responsible for regulating most (65 out of 82) of these miRNAs.

MiRNAs target the 3′UTR of mRNA and lead to translational arrest or degradation depending on the extent of base pairing in the ‘seed’ sequence. To throw light on the biological processes being regulated by the PHA-4-dependent miRNAs in *eat-2(ad1116)*, we predicted their target genes. Since the prediction of miRNA target is not always accurate, we used two different programs, miRanda as well as Targetscan (see Supporting information for references) and considered only the ones that came up with both the searches (Table S12). The two programs together predicted a total of 5145 possible unique targets of the 65 upregulated miRNAs (Table S13). We found that while *miR-71* had the largest number of predicted targets (782), *miR-243* has 34 targets. We also found that multiple miRNAs target the same mRNA (Table S13). For example, *syg-1* is targeted by 18 miRNA, *ptc-1* by 16, whereas *ima-3* and *ina-1* are targeted by 13 and 15 of the upregulated miRNA, respectively. SYG-1 is required cell autonomously in the HSNL neuron to determine synaptic specificity with vulval muscles and VC neurons [35]. PTC-1 is a *Drosophila* patched homolog, required for germline cytokinesis [36]. INA-1 is required during development for migration of neurons, coelomocyte precursor and distal tip cells of somatic gonad [37] while IMA-3 is the worm homolog of importin alpha involved oogenesis [38]. These genes may therefore be potentially important targets for miRNAs during DR.

Mature miRNAs, in most cases, bind to the 3′UTR of target mRNAs leading to their translational arrest [15]. Under this premise, we would expect to see down-regulation of protein levels corresponding to these predicted targets. In a previous study [39], the complete proteome of WT and *eat-2(ad1116)* were compared using quantitative proteomics. Out of the 167 proteins that were significantly downregulated in *eat-2(ad1116)*, we found that 74 of them are predicted targets of the miRNAs (hypergeometric analysis, *p=*2.67e-8; Table S14). This suggests that PHA-4-regulated miRNAs may translationally inhibit many of their target mRNAs.

### Many genes are upregulated in *eat-2(ad1116)* in a PHA-4-dependent manner

Next, in an effort to study the transcriptional response to DR, we compared the transcriptome of young-adult WT and *eat-2(ad1116)* worms collected on day 1 of adulthood, using NGS. We generated ~ 41 million reads for WT and ~42.2 million reads for *eat-2(ad1116)* using the GAIIx platform, as described in the experimental procedure. About 96.4% of WT reads and 97.2% of *eat-2(ad1116)* reads mapped to the *C. elegans* genome. We found that similar to the miRNA results, many more mRNAs were upregulated (3607 mRNAs) in *eat-2(ad1116)* compared to those that were downregulated (231 mRNAs)(Table S15).

Since PHA-4 plays such a central role in DR-mediated longevity, we asked whether these upregulated genes are under the direct control of PHA-4. For this, we looked for PHA-4 binding in the 5 kb promoter proximal region of the genes upregulated in *eat-2(ad1116)* using ChIPbase. We found that out of the 3607 upregulated transcripts, 2037 have at least one PHA-4 binding site (*p=*6.18e-171, by Hypergeometric test)(Table S15). To validate whether the presence of binding sites correlate with PHA-4 dependence in transcription, we randomly chose 10 genes. Using qRT-PCR, we found that 8 transcripts are dependent on PHA-4 (Figure 4B). These data suggests that PHA-4 may bind and upregulate a large number of genes in *eat-2(ad1116)*; this may be one of the reasons why knocking down PHA-4 completely suppresses DR-induced life span.

### Feed forward loops involving PHA-4 along with its target mRNA and miRNA

**Figure 5:**
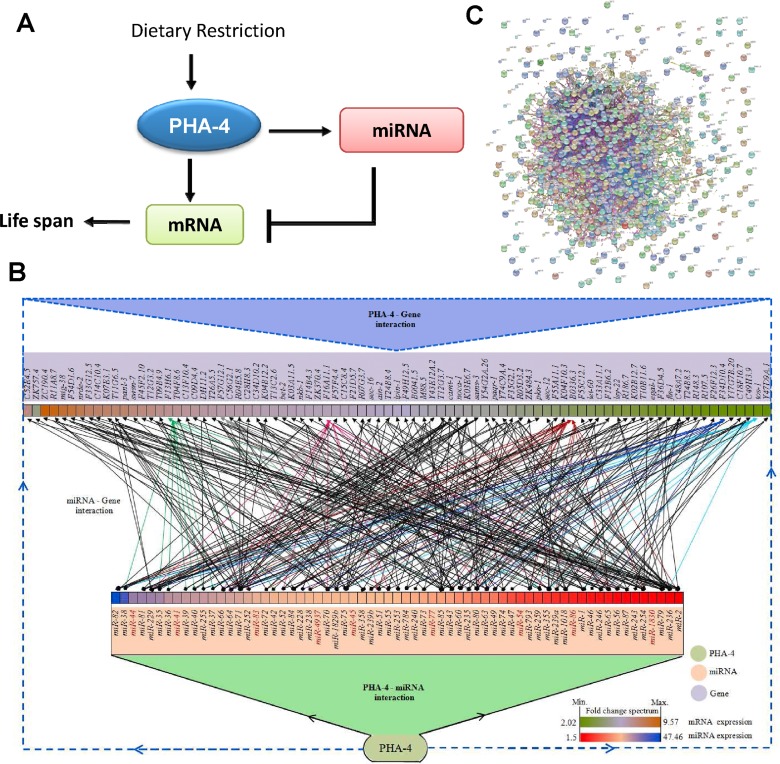
PHA-4 controls a large number of genes by feed-forward loops using miRNAs. (**A**) A representation of transcription factor-miRNA feed forward loop involving PHA-4 that controls life span during DR. (**B**) A complex regulatory network involving PHA-4 and its target mRNAs that are also regulated by PHA-4-controlled miRNAs. The network was generated using Cytoscape v3.1.1. Only a subset of genes targeted by PHA-4-regulated miRNA [as determined by mirTarBase were used to generate a representative decongested network. The boxes corresponding to the miRNA or mRNA/genes are colored based on their fold induction in **eat-2(ad1116)** as compared to WT. Edges are colored based on the number of miRNAs regulating a particular gene. Dark red are for genes regulated by 20 or more miRNAs while those regulated by less than 10 miRNAs are in black. MiRNAs that have no target listed in mirTarBase are in red. (**C**) The genes targeted by PHA-4 transcriptionally as well as post-transcriptionally are highly connected by protein-protein interactions, as determined by STRING software.

We have found that the FOXA transcription factor PHA-4 may be a critical requirement in upregulation of mRNAs as well as miRNA in *eat-2(ad1116)*. Next, we asked whether there was an overlap between genes that are transcriptionally upregulated by PHA-4 and the targets of PHA-4-regulated miRNA. By comparing 2037 PHA-4-regulated genes to 5145 genes that are predicted targets of PHA-4-regulated miRNA, we found a significant overlap (1073 genes with a *p* = 3.16e-176 by Hypergeometric test; Table S16). Thus, PHA-4 forms a large number of incoherent feed forward loops [40] during DR in *eat-2(ad1116)* (Figure 5A, B). While on one hand, PHA-4 transcriptionally upregulates the expression of a many transcripts, the same set of transcripts are potentially targeted by miRNAs that it transcribes (Figure 5B). In fact, some of the target genes are controlled by upto 20 miRNAs indicating that they might potentially play important roles during DR.

Next we asked whether the insulin-like signalling pathway also uses such FFLs to control gene expression. The insulin-like signalling pathway components phosphorylate and inactivate the FOXO transcription factor DAF-16 that is a major output of this cascade [32]. Under low insulin signalling conditions, such as in *daf-2(e1370)*, DAF-16 translocates into the nucleus and transactivates a large assortment of genes [32]. Using ChIPbase, we first determined that 22 of the 24 miRNA upregulated in *daf-2(e1370)* are potential direct targets of DAF-16. Next, using a published *daf-2(e1370)* transcriptomics data [41], we found that 687 transcripts are upregulated in *daf-2(e1370)*; out of these 336 have DAF-16 binding sites within 5 kb of promoter, according to ChIPbase. Thereafter, we overlapped the predicted targets of the 22 DAF-16-regulated miRNA (2235 transcripts) with that of the DAF-16-dependent mRNA. We found that only 47 genes overlapped (13.98%; *p=*0.01 by hypergeometric test) in contrast to that of *eat-2(ad1116)* where PHA-4 regulated 52.67% (*p* = 3.16e-176) of the upregulated transcripts by FFLs. This data suggested that PHA-4-mediated FFLs are a unique feature of DR-induced longevity in *eat-2(ad1116)* and may not be used extensively by DAF-16/FOXO downstream of the insulin-like signalling pathway.

### PHA-4 target genes regulated through FFLs control important biological functions

**Figure 6:**
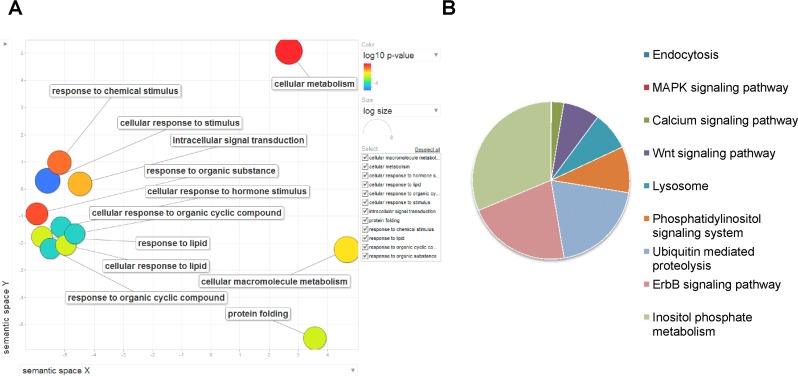
Genes regulated by PHA-4,transcriptionally as well as post-transcriptionally using miRNA are involved in important biological functions during DR. (**A**) The 1073 genes are enriched in GO terms pertaining to protein folding, cellular metabolism and response to external stimuli. GO analysis was performed using GORILA software and visualized with REVIGO. (**B**) KEGG pathway components for signaling, lysosome and ubiquitin-mediated decay are enriched in genes that are regulated by PHA-4-controlled FFLs.

Next we determined the functions of all the genes that are potentially transcriptionally as well as post-transcriptionally regulated by PHA-4. Since these genes are under such tight regulation, we can expect them to be important for DR-mediated longevity. We categorized these 1073 genes based on their Gene Ontology (GO) terms as well as determined which molecular pathways these genes function in. We used “Gene Ontology enRIchment anaLysis and visualization” tool (GOrilla) and visualized the results using Revigo. We found that the GO terms associated with metabolism, protein folding and response to various chemical stimuli are enriched (Figure 6A). These genes are involved in ubiquitin mediated protein degradation, lysosomal autophagy, inositol phosphate metabolism, phosphatidylinositol signalling, ErbB signalling as determined by KEGG pathway analysis using DAVID (see Supporting information for references) (Figure 6B). These 1073 genes are also highly connected with each other through protein-protein interaction as determined by STRING database (Figure 5C). Further, these genes are enriched for transcription factors (~10% of the 1073 genes code for transcription factors as against 4.4% found in the genome; *p*=1.68e-12 by Hypergeometric test). Interestingly, when we compared this list of genes with the GeneAge database (http://genomics.senescence.info/genes/), we found that 86 genes are already known to be important in longevity determination having *p=*1.66e-12 (Table 2). Together, the genes that are regulated by PHA-4 directly or indirectly through miRNAs most likely are important players in DR-mediated longevity in *eat-2(ad1116)*.

**Table 2:** List of aging-associated genes that are transcriptionally as well as post-transcriptionally regulated by PHA-4

Gene Name	Effect on lifespan	Functions
*acdh-13*	Increase	Acyl CoA DeHydrogenase
*akt-1*	Increase	AKT kinase family
*aps-1*	Increase	AdaPtin, Small chain (clathrin associated complex)
*bec-1*	Decrease	BEClin (human autophagy) homolog
*C47D12.2*	Increase	-
*C56G2.1*	Increase	KH domain-containing protein
*ccr-4*	Decrease	CCR (yeast CCR4/NOT complex component) homolog
*cec-3*	Increase	Chromo domain-containing protein
*ceh-20*	Decrease	Homeobox containing protein
*chc-1*	Decrease	Clathrin Heavy Chain
*cua-1*	Decrease	CU (copper) ATPase
*daf-12*	Increase	A member of the steroid hormone receptor superfamily
*daf-14*	Increase	Possibly a transducer of the DAF-7/TGF-beta-mediated signal that promotes reproductive growth
*daf-16*	Decrease	Forkhead family transcription factor
*daf-3*	Decrease	Transcriptional regulator
*daf-4*	Increase	Transmembrane serine/threonine kinase
*din-1*	Decrease	DAF-12 Interacting Protein
*dlk-1*	Decrease	DAP (Death Associated Protein kinase) Like Kinase
*dpy-27*	Decrease	ATP-binding protein that is a homolog of the SMC4 subunit of mitotic condensin
*dve-1*	Decrease	DVE (Defective proVEntriculus in Drosophila) homolog
*egl-18*	Increase	GATA family transcription factor
*egl-27*	Increase	Homolog of human MTA1 (metastasis-associated protein), a part of a nucleosome remodeling and histone deacetylation (NURD) complex
*egl-8*	Increase	phospholipase C beta
*egl-9*	Increase	Proline hydroxylase
*epc-1*	Increase	Enhancer of PolyComb-like
*erm-1*	Increase	Ezrin/Radixin/Moesin
*ets-4*	Increase	ETS class transcription factor
*F09F7.5*	Increase	-
*F14B4.3*	Increase	DNA-directed RNA polymerase
*gcy-28*	Increase	Guanylyl CYclase
*gei-4*	Decrease	GEX Interacting protein
*hpk-1*	Decrease	Homeodomain interacting Protein Kinase
*ire-1*	Decrease	IRE1 kinase related
*itr-1*	Increase	Inositol Triphosphate Receptor
*klo-2*	Decrease	-
*kri-1*	Decrease	human KRIT 1 (Krev interaction trapped/cerebral cavernous malformation 1) homolog
*ldb-1*	Decrease	LIM domain Binding Protein
*let-23*	Increase and Decrease	EGF-receptor-family transmembrane tyrosine kinase
*let-502*	Decrease	Rho-binding Ser/Thr kinase, orthologous to human myotonic dystrophy kinase (DM-kinase)
*let-60*	Increase	Member of the GTP-binding RAS protooncogene family
*lin-15B*	Decrease	Novel protein that contains a THAP domain, a zinc-coordinating, site-specific DNA-binding domain
*lin-23*	Decrease	F-box- and WD-repeat-containing protein, components of SCF (Skp1, Cullin, F-box) ubiquitin-ligase complexes that function in ubiquitin-mediated protein degradation
*lin-3*	Decrease	EGF family of peptide growth factors
*lin-5*	Increase	-
*max-2*	Increase	Motor AXon guidance
*mep-1*	Decrease	Mog interacting, Ectopic P granules
*mfn-1*	Increase	Mitoferrin
*mnk-1*	Decrease	MAP kinase iNtegrating Kinase (MNK) homolog
*mpz-2*	Increase	Temporarily Assigned Gene name
*mrp-5*	Increase	Multidrug Resistance Protein family
*mtm-3*	Decrease	MTM (myotubularin) family
*nac-2*	Increase	NADC (Na+-coupled dicarboxylate transporter) family
*nfi-1*	Decrease	Transcription/replication factor
*nhr-14*	Increase	Nuclear Hormone Receptor family
*nhr-23*	Increase	Nuclear Hormone Receptor family
*nhr-49*	Decrease	Nuclear Hormone Receptor family
*nhx-2*	Increase	Na/H eXchanger
*nkcc-1*	Increase	Na-K-Cl Cotransporter homolog
*npa-1*	Increase	Fatty acids and vitamin A binding
*pab-2*	Increase	PolyA Binding protein
*pup-2*	Increase	Poly(U) Polymerase
*R08E3.3*	Increase	-
*rab-5*	Decrease	RAB family
*rbr-2*	Decrease	RB (Retinoblastoma Binding protein) Related
*rpa-1*	Increase	Replication Protein A homolog
*sel-5*	Increase	Suppressor/Enhancer of Lin-12
*set-2*	Increase	SET (trithorax/polycomb) domain containing
*set-6*	Decrease	SET (trithorax/polycomb) domain containing
*sgk-1*	Increase	Serum- and Glucocorticoid-inducible kinase homolog
*smg-1*	Increase	Suppressor with Morphological effect on Genitalia
*smk-1*	Decrease	SMEK (Dictyostelium Suppressor of MEK null) homolog
*syd-2*	Decrease	Required cell autonomously in neurons for differentiation of presynaptic active zones
*tax-6*	Increase	Required for inhibition and adaptation of several sensory neurons and for the normal regulation of egg-laying by serotonin
*tes-1*	Increase	Temporarily Assigned Gene name
*top-1*	Decrease	TOPoisomerase
*unc-26*	Increase	Encodes synaptojanin, a polyphosphoinositide phosphatase
*unc-32*	Increase	Vacuolar H+-ATPase V0 sector, subunit a
*unc-52*	Increase	Basement-membrane (HSPG) core
*unc-62*	Increase	Meis-class homeodomain protein
*unc-76*	Increase	Encodes FEZ family protein involved in axon-axon interactions
*wdr-23*	Increase	WD Repeat protein
*wip-1*	Decrease	Wiskott-Aldrich syndrome protein (WASP)-Interacting Protein and gene assignment
*wts-1*	Increase	WarTS (Drosophila) protein kinase homolog
*wwp-1*	Decrease	WW domain Protein (E3 ubiquitin ligase)
*Y48G1A.4*	Increase	Ribosome biogenesis
*zfp-1*	Decrease	leucine zipper, zinc finger, and PHD/LAP domain protein

## DISCUSSION

Although the role of DR in life span regulation in *C. elegans* is well-established, the underlying molecular mechanisms are only now beginning to be elucidated. The role of the FOXA transcription factor PHA-4 in DR was identified several years ago, but very little is known about its functional regulation during DR [11]. Initial studies had indicated that PHA-4 levels increase during DR in *eat-2(ad1116)* and the transcription factor controls genes involved in oxidative stress resistance (like *sod-2* and *sod-4*) [11] or autophagy [42]. In this study, we show for the first time the complexity of gene regulation brought about by this transcription factor. We found that PHA-4 upregulates a large contingent of genes in *eat-2(ad1116)*; however, it also targets these genes using miRNAs that it controls, forming incoherent FFLs. There are not many examples of such large sets of TF-miRNA FFLs in biology, although most of the miRNA have been predicted to function in this manner [43]. Such intricate mechanisms of gene expression control may make PHA-4 a major factor in DR-induced life span regulation. Perturbation of PHA-4 may destabilize the system during DR, leading to complete suppression of life span, as observed earlier [11].

PHA-4 is a central part of a large number of incoherent feed forward loops (Figure 5B), as evident from our study. A miRNA would bind to the 3′UTR of its target mRNA to inhibit translation or in some cases, lead to its destabilization and degradation [14-16]. One of the main objectives of such miRNA-mRNA interaction is to shield protein expression from fluctuations in transcription [17-20]. This mechanism also has a built-in rheostat that ensures that translation of a protein will effectively proceed only when the expression of mRNA picks up, titrating away the miRNA inhibition [21]. Increased noise in gene expression would lead to phenoypic variability during development or environmental perturbations, a process that is curbed by canalization or robustness in the system [21]. Thus, PHA-4 may be the master regulator of gene expression that is responsible to instil robustness in the system, reducing phenotypic variability during DR. By controlling a large number of miRNAs and mRNAs in from of incoherent feed-forward loops, the transcription factor also ensures an appropriate DR response only when nutrition levels fall to a critical level. This may effectively save a lot of cellular energy by preventing the system to mount an all-out defence against nutritional deprivation when there are only minor fluctuations in food availability.

The genes that are intensely protected from expression fluctuations by PHA-4 may constitute important components of the DR response in *eat-2(ad1116)*. When we analysed the 1073 genes that are regulated by PHA-4 transcriptionally as well as post-transcriptionally, we found them to be categorized in KEGG pathways for endocytosis, lysosome-mediated autophagy, cellular signalling and ubiquitin-mediated proteolysis. These genes were also enriched for GO terms pertaining to intracellular signalling and cellular response to organic compounds including lipids, hormones etc., as well as protein folding.

It appears that genes involved in protein turnover, under the transcriptional control of PHA-4, are particularly targeted by the FFLs. Ubiquitin-mediated proteosomal degradation involving ubiquitin ligases is a well-known requirement for DR-mediated longevity as well as for starvation response [44-47]. The HECT (*H*omologous to *E*6AP *C*arboxy *T*erminus) E3 ubiquitin ligase WWP-1 is required for DR-induced life span extension while components of a SCF E3 ubiquitin ligase have been shown to be important for fasting-induced longevity [44, 46]. In fact, our data suggests that *wwp-1* is one of the targets of PHA-4 that is also regulated by miRNA. Similarly, Skp1-Cullin-F-box (SCF) E3 ligase complex genes like *skr-1*, *cul-3*, *cul-4*, *cul-5, lin-23* as well as Ubiquitin conjugating enzyme *ubc-25* are also regulated through the PHA-4-dependent FFLs. The WD40 repeat protein WDR-23 along with the ubiquitin ligase CUL4/DDB1 functions to regulate the SKN-1/NRF2 transcription factor [48]. SKN-1 activity in the neurons is required for DR-mediated longevity [49]. We found that the *wdr-23* and *cul-4* genes are regulated by PHA-4 FFLs. Additionally, components of the lysosome-mediated autophagy are also targeted by FFLs. Beclin1 homolog in worms *bec-1* is the major regulator of autophagy, a process that requires PHA-4 activity [50]. We found that *bec-1* as well as other autophagic genes like *atg-2* (yeast atg2p and human ATG2A/ATG2B homolog) and *atg-11* (fly Atg17-PC homolog) may be regulated transcriptionally as well as post-transcrip-tionally by PHA-4, Thus, protein turnover that provides building blocks for synthesis of new proteins and fuel cell with energy during energy-deprived conditions are primary contributors of longevity [47] and thus may be under tight regulatory control by PHA-4.

Signal transduction pathways are other important components of DR that seems to be insulated from fluctuations by PHA-4 FFLs. Components of MAP kinase signalling, calcium signalling and Wnt signalling are enriched. Signalling components may play critical role during DR to maintain cellular homeostasis and tight control of their protein expression is required to minimize phenotypic fluctuations. Well known signal transduction components, like *akt-1*and *sgk-1*(mammalian AKT and SGK homologs), *tir-1*(Toll-like receptor homolog), *sek-1* (MKK4 homolog that acts in the p38 pathway), *pkc-1* (mammalian protein kinase C epsilon homolog), *nipi-3* (the human kinase Tribbles homolog), *mbk-1* (orthologous to Drosophila MINIBRAIN and mammalian DYRK1A/MnbK), *jun-1* (mammalian JUN homolog), *let-60* (member of the GTP-binding RAS protooncogene family) etc. are potentially regulated by the FFLs; these genes include ones with known role in aging and stress. Interestingly, transcription factor genes are also highly enriched in the genes regulated by FFLs. The system may be protecting these important transcription factors from gene expression fluctuations in order to maintain proper cellular functions during DR, leading to increased life span. In fact, the genes that are part of the FFLs downstream of PHA-4 are significantly enriched for genes that are involved in life span regulation.

While this study was in review, Smith-Vikos et al. [51] reported PHA-4 as the most well-connected factor in a transcription factor-miRNA regulatory network involved in aging. They identified two miRNAs that are potentially directly regulated by PHA-4 and are upregulated during two different DR interventions, bacterial dilution and in *eat-2(ad1116)*. We also found that these two miRNA were significantly upregulated in our study, although to a greater extent. This difference may be attributed to the stages at which the worms were harvested for analysis. Together, both the studies attest to the central role of PHA-4 in controlling miRNA expression during DR to regulate life span.

In conclusion, our study reveals the complexity of gene regulation during DR in *eat-2(ad1116)*, showing PHA-4 as a central regulator of gene expression that may infuse robustness in the system using a large number of FFLs to protect against phenotypic variations during periods of low food availability. It will be interesting to study whether alternative models of DR in worms or other organisms have similar post-transcriptional regulation by FOXA transcription factors.

## METHODS

Complete and detailed experimental procedure is available as Supporting information.

### Strain maintenance

All strains were maintained at 20°C using standard *C. elegans* techniques. All RNAi experiments were initiated using synchronized L1 stage worms. Strains used in the study are: N2 Bristol as WT, *eat-2(ad1116), rrf-3(pk1246), eat-2(ad1116);rrf-3(pk1246), alg-1(gk214)* and *alg-2(ok304)*.

For collecting worms for NGS analysis, synchronized L1 larvae were placed on Nematode Growth Media (NGM) agar plates and allowed to grow till L4 stage. The plates were overlaid with FuDR to stop the hatching of the eggs. Worms were collected from the plates by washing with 1 X M9 the following day (day 1/young-adult worms) or on day 8 (aging worms). The worm pellet was washed three times with 1 X M9 before Trizol reagent (Invitrogen, USA) was added and RNA isolated as described below.

### RNA isolation

RNA isolation was performed using Trizol. Briefly, worms grown on OP50 bacteria, vector or RNAi of interest were washed off the plates with M9 buffer. Thereafter, 0.4 ml of Trizol reagent was added and the worms lysed by vigorous vortexing. RNA was purified by phenol:chloroform:isoamylalcohol extraction and ethanol precipitation. RNA integrity were confirmed by analysis on an Agilent 2100 bioanalyzer, using the RNA 6000 nano kit (Agilent) to confirm that the RIN numbers are above 8.0. Alternatively, the quality of the ribosomal RNA 28 S and 18 S as determined on an agarose gel was used as a measure of integrity and the absorbance at 260/280 nm was used to determine quantity.

RNA library preparation, Next Generation sequencing and data analysis. The small RNA libraries were constructed using Small RNA sample preparation kit v1.5 according to the manufacturer's instructions (Illumina Inc., USA). Briefly, the total RNA (2μg) were ligated to 3′ RNA adapter using RNA ligase truncated and 5′ adapter using T4 RNA ligase 2 (New England Biolabs, USA). The ligation products were reverse transcribed using Superscript II Reverse Transcriptase (Life Technologies, USA) and amplified with 12 cycles of PCR. The PCR products constituting the small RNA cDNA libraries were resolved on 6% Novex TBE PAGE Gel (Invitrogen, USA) and ~150 bp fragments excised. The library was eluted from the acrylamide gel and analyzed on Agilent 2100 Bioanalyzer using DNA high sensitivity kit (Agilent Technologies, USA). NGS of cDNA libraries were performed using Illumina GA*II_X_* for 36 cycles. A total of 2.8 GB of raw sequence data, comprising of WT and *eat-2(ad1116)* strains at both day 1 and day 8, was imported into the CLC Genomics Workbench 6.5.1 (CLC Bio, Denmark). The reads were trimmed off the adapter sequences and reads containing low quality bases eliminated. The trimmed raw sequences were mapped to the miRBase release 19, allowing for a maximum of two gaps or mismatches. Unpaired group comparisons, based on Transcript Per Million (TPM), were used as expression values to compare different samples. A fold change ±1.5 with a minimum read count of ≥10 were used to filter the differentially expressed miRNA. The *p* value cutoff was set at *p*≤0.05 based on Kal's Z test statistical. The sequencing data is available at GEO repository with Series record number GSE60155 and GSE61112.

RNA-Sequencing (RNA-seq) libraries of WT and *eat-2(ad1116)* Day 1 samples were prepared as recommended by the Illumina TruSeq™ RNA Sample Preparation kit using Low-Throughput (LT) Protocol (Illumina, Inc.,USA). NGS of libraries was performed using Illumina GA *II_X_* for 78 cycles including 6 additional cycles for index read. Sequence reads were aligned using CLC Genomics Workbench 6.5.1 with default setting against *C. elegans* genome assembly (WS231). Unpaired group comparisons, based on RPKM (Reads Per Kilobase per Million mapped reads), were chosen as expression values for comparing the samples. A fold change ±2.0 and *P* value ≤0.05 (Kal's Z test) were used to filter the differentially expressed genes.

## SUPPLEMENTAL DATA FIGURES AND TABLES




