# Supplementary material for: PHA-4/FOXA-regulated microRNA feed forward loops during Caenorhabditis elegans dietary restriction
Source: Aging (Albany NY). 2014 Oct 31;6(10):835–51. doi: 10.18632/aging.100697 (PMC4247386; doi:10.18632/aging.100697)
Supplement: Supplementary file 1 [file aging-06-835-s001.pdf]

SUPPLEMENTAL DATA

Detailed Experimental Procedures

Strain maintenance. All strains were maintained at 20°C using standard *C. elegans* techniques (Stiernagle 2006). All RNAi experiments were initiated using synchronized L1 stage worms. Strains used in the study are: N2 Bristol as wild-type, *eat-2(ad1116), rrf-3(pk1246), eat-2(ad1116);rrf-3(pk1246), alg-1(gk214)* and *alg-2(ok304).*

For collecting worms for Next generation sequencing analysis, synchronized L1 larvae were placed on Nematode Growth Media (NGM) agar plates and allowed to grow till L4 stage. The plates were overlaid with FuDR to stop the hatching of the eggs. Worms were collected from the plates by washing with 1 X M9 the following day (day 1/young-adult worms) or on day 8 (aging worms). The worm pellet was washed three times with 1 X M9 before Trizol reagent (Invitrogen, USA) was added and RNA isolated as described below.

Preparation of RNAi plates. For preparing RNAi plates, NGM media was supplemented with 100 µg/ml ampicillin and 2 mM IPTG. RNAi bacteria were grown overnight at 37°C in LB media that was supplemented with 100 µg/ml ampicillin and 12.5 µg/ml tetracycline. The following day, the cultures were diluted (1:50) in LB containing 100 µg/ml ampicillin and grown at 37°C until an OD_{600} of 0.6. The pelleted bacterial cells were resuspended in 1X M9 buffer containing 1 mM IPTG and 100 µg/ml ampicillin. About 200 µl of the bacterial suspension was seeded onto the RNAi plates which were dried at room temperature for 2-3 days and stored at 4°C till further use.

RNA isolation. RNA isolation was performed using Trizol. Briefly, worms grown on OP50 bacteria, vector or RNAi of interest were washed off the plates with M9 buffer. Thereafter, 0.4 ml of Trizol reagent was added and the worms lysed by vigorous vortexing. RNA was purified by phenol:chloroform:isoamylalcohol extraction and ethanol precipitation. RNA integrity were confirmed by analysis on an Agilent 2100 bioanalyzer, using the RNA 6000 nano kit (Agilent) to confirm that the RIN numbers are above 8.0. Alternatively, the quality of the ribosomal RNA 28 S and 18 S as determined on an agarose gel was used as a measure of integrity and the absorbance at 260/280 nm was used to determine quantity.

RNA library preparation, Next Generation sequencing and data analysis. The small RNA libraries were constructed using Small RNA sample preparation kit v1.5 according to the manufacturer's instructions (Illumina Inc., USA). Briefly, the total RNA (2µg) were ligated to 3' RNA adapter using RNA ligase truncated and 5' adapter using T4 RNA ligase 2 (New England Biolabs, USA). The ligation products were reverse transcribed using Superscript II Reverse Transcriptase (Life Technologies, USA) and amplified with 12 cycles of PCR. The PCR products constituting the small RNA cDNA libraries were resolved on 6% Novex TBE PAGE Gel (Invitrogen, USA) and ~150 bp fragments excised. The library was eluted from the acrylamide gel and analyzed on Agilent 2100 Bioanalyzer using DNA high sensitivity kit (Agilent Technologies, USA). Next generation sequencing of cDNA libraries were performed using Illumina GAII_X for 36 cycles. A total of 2.8 GB of raw sequence data, comprising of WT and *eat-2(ad1116)* strains at both day 1 and day 8, was imported into the CLC Genomics Workbench 6.5.1 (CLC Bio, Denmark). The reads were trimmed off the adapter sequences and reads containing low quality bases eliminated. The trimmed raw sequences were mapped to the miRBase release 19, allowing for a maximum of two gaps or mismatches. Unpaired group comparisons, based on Transcript Per Million (TPM), were used as expression values to compare different samples. A fold change ±1.5 with a minimum read count of ≥10 were used to filter the differentially expressed miRNA. The *p* value cutoff was set at $p \leq 0.05$ based on Kal's Z test statistical analyses (Kal *et al.* 1999). The sequencing data are available at GEO repository with Series record number GSE60155.

RNA-Sequencing (RNA-seq) libraries of WT and *eat-2(ad1116)* Day 1 samples were prepared as recommended by the Illumina TruSeq™ RNA Sample Preparation kit using Low-Throughput (LT) Protocol (Illumina, Inc.,USA). Next Generation sequencing of libraries was performed using Illumina GA II_X for 78 cycles including 6 additional cycles for index read. Sequence reads were aligned using CLC Genomics Workbench 6.5.1 with default setting against *C. elegans* genome assembly (WS231). Unpaired group comparisons, based on RPKM (Reads Per Kilobase per Million mapped reads), were chosen as expression values for comparing the samples. A fold change ±2.0 and *P* value ≤0.05 (Kal's Z test) were used to filter the differentially expressed genes.

Novel miRNA prediction. Unannotated reads of both WT and *eat-2(ad1116)* on Day 1 and Day 8 were used for novel miRNA prediction. For this purpose miRDeep2 program, running on default parameters (Friedlander *et al.* 2012), was used. All pre-requisite data necessary for running miRDeep2 pipeline were downloaded from miRBase release 19.

Target Gene Prediction. To identify the potential targets of each differentially regulated miRNA, we used two different target gene prediction algorithms, miRanda (John *et al.* 2004) and TargetScan (Lewis *et al.* 2005). Targets were predicted based on seed matching, cross-species conservation, and targeted pair energy. Targets commonly predicted by both algorithms were used for further analysis.

qRT-PCR analysis. To validate the expression of mature miRNA, cDNA was synthesized from DNase-treated total RNA using the Universal cDNA Synthesis Kit II (Exiqon Inc., Denmark). The reaction was incubated in a thermocycler at 42 ^{o}C for 60 min, heat-inactivated at 95 ^{o}C for 5 min and then cooled to 4 ^{o}C. Primers for each mature miRNA was designed according to previously published literature (Balcells *et al.* 2011). To detect expression of mRNA transcripts, cDNA was synthesized from 2.5 µg of DNase-treated total RNA using the SuperScript III cDNA synthesis kit (Invitrogen, USA). MiRNA and mRNA expression levels were determined by quantitative real time PCR (qRT-PCR) using the Mesagreen MasterMix (Eurogentec, Belgium) and Realplex PCR system (Eppendorf, USA). Relative gene expression levels were calculated based on ΔΔCT method (Schmittgen & Livak 2008). Statistical analysis was performed using SigmaPlot 10.0 (Systat software, USA).

Databases Used in this Study and their References

ChIPBase- (Yang *et al.* 2013)
miRanda- (John *et al.* 2004)
Targetscan- (Lewis *et al.* 2005)
Gorilla- (Trikka *et al.* 2002)
Revigo- (Supek *et al.* 2011)
DAVID (Dennis *et al.* 2003)
mirTarBase (Hsu *et al.* 2011)
STRING database (Jensen *et al.* 2009; Franceschini *et al.* 2013)
miRDeep2 (Friedlander *et al.* 2012)

References

1. Balcells I, Cirera S, Busk PK. Specific and sensitive quantitative RT-PCR of miRNAs with DNA primers. BMC biotechnology.2011; 11:70.
2. Dennis G Jr, Sherman BT, Hosack DA, Yang J, Gao W, Lane HC, Lempicki RA.DAVID: Database for Annotation, Visualization, and Integrated Discovery. Genome Biol. 2003; 4:P3.
3. Franceschini A, Szklarczyk D, Frankild S, Kuhn M, Simonovic M, Roth A, Lin J, Minguez P, Bork P, von Mering C , Jensen LJ. STRING v9.1: protein-protein interaction networks, with increased coverage and integration. Nucleic acids research. 2013; 41:D808-815.
4. Friedlander MR, Mackowiak SD, Li N, Chen W , Rajewsky N miRDeep2 accurately identifies known and hundreds of novel microRNA genes in seven animal clades. Nucleic acids research. 2012; 40:37-52.
5. Hsu SD, Lin FM, Wu WY, Liang C, Huang WC, Chan WL, Tsai WT, Chen GZ, Lee CJ, Chiu CM, Chien CH, Wu MC, Huang CY, Tsou AP, Huang HD. miRTarBase: a database curates experimentally validated microRNA-target interactions. Nucleic acids research. 2011; 39:D163-169.
6. Jensen LJ, Kuhn M, Stark M, Chaffron S, Creevey C, Muller J, Doerks T, Julien P, Roth A, Simonovic M, Bork P , von Mering C . STRING 8--a global view on proteins and their functional interactions in 630 organisms. Nucleic acids research. 2009; 37:D412-416.
7. John B, Enright AJ, Aravin A, Tuschl T, Sander C , Marks DS. Human MicroRNA targets. PLoS Biol. 2004; 2:e363.
8. Kal AJ, van Zonneveld AJ, Benes V, van den Berg M, Koerkamp MG, Albermann K, Strack N, Ruijter JM, Richter A, Dujon B, Ansorge W , Tabak HF. Dynamics of gene expression revealed by comparison of serial analysis of gene expression transcript profiles from yeast grown on two different carbon sources. Molecular biology of the cell. 1999; 10:1859-1872.
9. Lewis BP, Burge CB , Bartel DP. Conserved seed pairing, often flanked by adenosines, indicates that thousands of human genes are microRNA targets. Cell. 2005; 120:15-20.
10. Schmittgen TD , Livak KJ. Analyzing real-time PCR data by the comparative C(T) method. Nature protocols.2008; 3:1101-1108.
11. Stiernagle T (2006). Maintenance of C. elegans. WormBook, 1-11.
12. Supek F, Bosnjak M, Skunca N , Smuc T. REVIGO summarizes and visualizes long lists of gene ontology terms. PLoS One. 2011; 6:e21800.
13. Trikka D, Fang Z, Renwick A, Jones SH, Chakraborty R, Kimmel M , Nelson DL. Complex SNP-based haplotypes in three human helicases: implications for cancer association studies. Genome research. 2002; 12:627-639.
14. Yang JH, Li JH, Jiang S, Zhou H , Qu LH. ChIPBase: a database for decoding the transcriptional regulation of long non-coding RNA and microRNA genes from ChIP-Seq data. Nucleic acids research. 2013; 41:D177-187.

Supplemental Figures

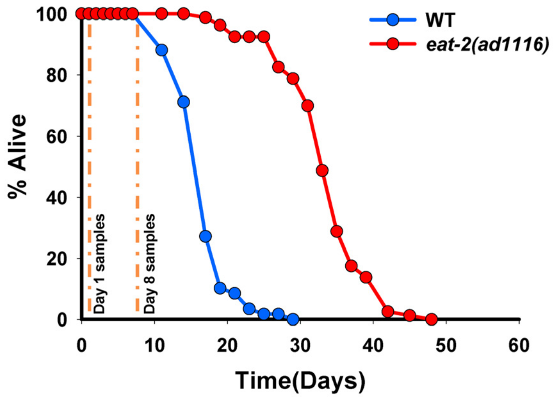

Strain	N	MLS ± SEM	*p* value
WT	59	16.83 ± 0.45	
eat-2(ad1116)	80	33.50 ± 0.67	<.0001

Figure S1. Lifespan of WT and *eat-2(ad1116)* worms. RNAs collected at Day 1 and Day 8, as highlighted in lifespan, were used for miRNA library preparation and Next Generation sequencing. No significant death was observed at these time points

A

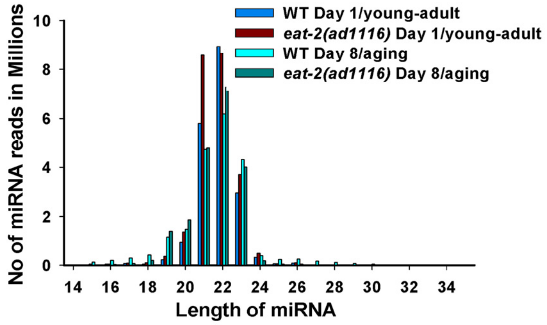

B

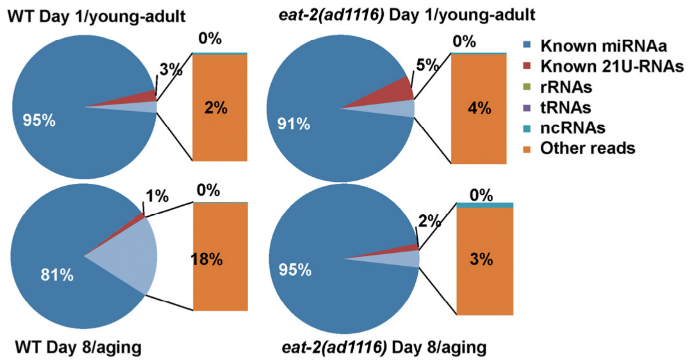

Figure S2. Next Generation sequencing of miRNAs from WT and *eat-2(ad1116)*. (**A**) Adapter-trimmed sequences were enriched for ~22 nucleotide RNA molecules. (**B**) Distribution of different classes of small RNAs including known miRNA, 21U-RNAs, rRNAs, tRNAs and noncoding RNA species. The 'other' reads contain sequences that mapped to the genome, but not to any known RNA. These were used for novel miRNA discovery.

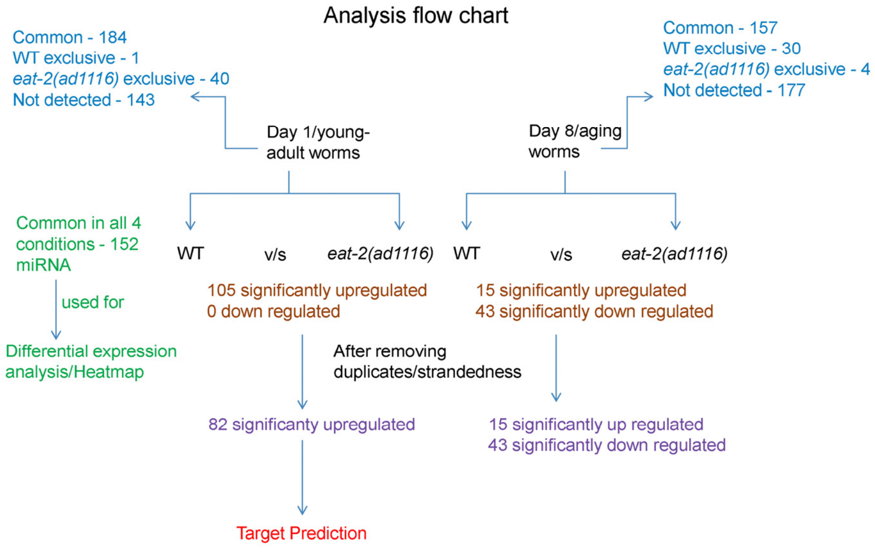

Figure S3. Flowchart and brief results of analysis

Supplemental Tables

Please follow the links in Full Text version of this manuscript to see the Supplemental Tables listed below:

Table S1- Sequencing details and mapping summary for small RNA sequencing for Wild-type (WT) and *eat-2(ad1116)*
Table S2: Comparison of miRNA expression between this study and that reported by Kato et al., 2011
Table S3: MiRNAs that changed expression significantly in *eat-2(ad1116)* as compared to WT in young-adult (Day 1) and aging (Day 8) worms
Table S4: MiRNA exclusively expressed in young-adult *eat-2(ad1116)* worms collected on Day 1
Table S5: MiRNA exclusively expressed in aging WT or *eat-2(ad1116)* worms collected on Day 8
Table S6: MiRNA expression pattern in young-adult or aging WT and *eat-2(ad1116)* worms
Table S7: List of 79 miRNAs that expressed in one or more of the 4 samples (young-adult or aging WT and *eat-2(ad1116)*, but not in all
Table S 8: Predicted novel miRNA in *eat-2(ad1116)*
Table S 9: Predicted novel miRNA in WT
Table S 10: Regulation of novel miRNA that are common to *eat2(ad1116)* and WT
Table S 11: Details of 3 selected novel miRNA candidate used for validation
Table S12: The number of targets predicted bioinformatically for miRNAs upregulated in young-adult *eat-2(ad1116)* worms collected on day 1 of adulthood
Table S13: Identity of genes predicted to be targets of miRNAs upregulated in young-adult (day 1) *eat-2(ad1116)* worms
Table S14: List of predicted miRNA target genes whose proteins levels are downregulated in *eat-2(ad1116)*
Table S15: Differentially expressed mRNA in young-adult *eat-2(ad1116)* as compared to WT
Table S16: Genes that are regulated transcriptionally as well as post-transcriptionally by PHA-4
Table S17: List of primers used in this study
Table S18: MiRNA up- or downregulated in *daf-2(e1370)* compared to WT in young adult/day 1 worms
